# Sentinel Lymph Node Mapping: Current Applications and Future Perspectives in Gynecology Malignant Tumors

**DOI:** 10.3389/fmed.2022.922585

**Published:** 2022-06-29

**Authors:** Tianyou Wang, Yan Xu, Wenyu Shao, Chao Wang

**Affiliations:** Department of Obstetrics and Gynecology, Obstetrics and Gynecology Hospital, Fudan University, Shanghai, China

**Keywords:** sentinel lymph node, mapping, cervical cancer, ovarian cancer, endometrial cancer, vulvar cancer, gynecological cancer, clinical trials

## Abstract

The sentinel lymph nodes (SLNs) is a group of lymph nodes initially involved in the metastatic spread of cancer cells. SLN mapping refers to intraoperative localization and biopsy of SLNs with specific tracers to assess lymph node metastases. It is widely used in a variety of tumor surgeries for its high sensitivity and high negative predictive value. In the evaluation of the status of lymph node metastases in gynecological malignancies, it has received increasingly more attention due to its minor invasiveness, few complications, and high diagnosis rate. The National Comprehensive Cancer Network (NCCN) guidelines provide an excellent introduction to the indications and methods of SLN techniques in vulvar, cervical, and endometrial cancers, but they provide little explanation about some specific issues. In this review, we summarize different dyes and injection methods and discuss the indications of application and the clinical trials of SLN mapping in gynecological malignant tumors, aiming to provide a reference for the rational application of sentinel techniques in gynecology malignant tumors before relevant guidelines are updated.

## Introduction to Sentinel Lymph Node Techniques

### Gynecologic Tumors: From Lymph Node Dissection to Sentinel Lymph Node Mapping

**FIGURE 1:**
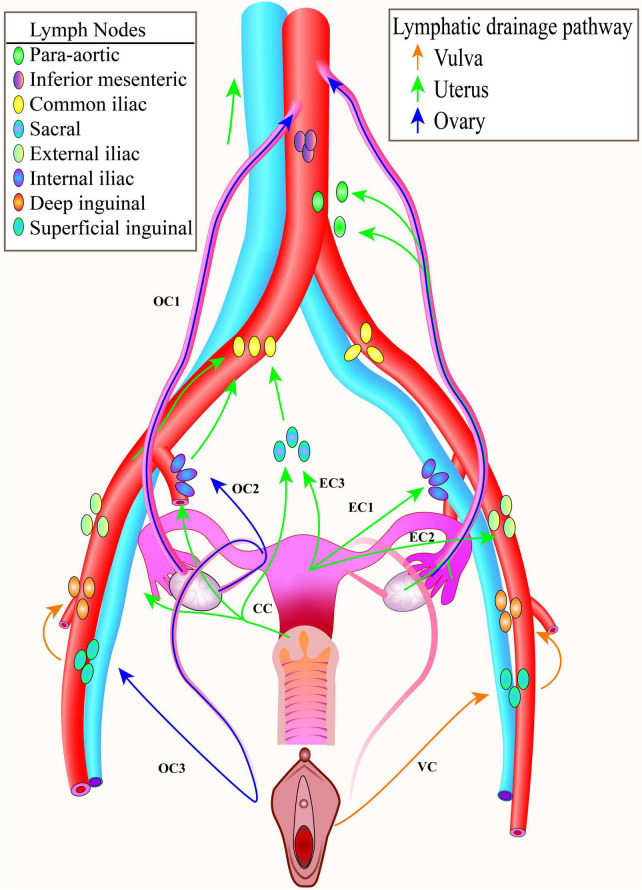
Pelvic lymphatic drainage. The common lymphatic drainage pathways of the ovary, the uterus, the cervix, the vagina and the vulva. To facilitate display, only one side lymphatic drainage route was drawn for each tumor. CC, cervical cancer; EC, endometrial cancer; OC, ovarian cancer; VC, vulva cancer.

Among the top 10 malignancies in women, ovarian cancer ranks 5th, with an estimated 12,810 new deaths, and uterine corpus cancer ranks 6th, with an estimated 12,550 new deaths (1). Gynecological malignant tumors seriously threaten women’s health, and the prognosis of patients is closely related to the staging of the disease. When lymph node metastasis occurs, it is usually stage III or above, which belongs to the category of advanced stage and has a poor prognosis. The assessment status of lymph node metastasis not only involves the evaluation content of the tumor stage but also provides the basis for the formulation of individual treatment plans. About 30 years ago, systematic lymph node dissection (Figure 1) was commonly used for gynecologic malignancies (2) to reduce the tumor cell load and remove all lymph nodes in the drainage area of the organ where the tumor was located for pathological biopsy. However, this method is highly damaging and takes a long time to operate, which destroys the original lymphatic drainage path and often leads to complications such as lymphedema (morbidity: 32.1%, endometrial carcinomas) (3, 4), lymphocytic cysts (54.3%, vulva cancer), cellulitis (41.3%, vulva cancer), and wound dehiscence (36.2%, vulva cancer) (5). In recent years, with the advent of minimally invasive surgery and accelerated rehabilitation concepts, the scope of surgical resection has gradually narrowed. The lymphatic mapping and SLN biopsy technology were developed to reduce the incidence of postoperative complications and reduce unnecessary lymph node resection in non-metastatic patients and preserve the function of lymphatic ducts.

### Definition of Sentinel Lymph Nodes

Sentinel lymph nodes refer to the first group of lymph nodes that drain the primary tumor lymph, which is the first area involved when the tumor metastasis occurs, reflecting the state of lymph node involvement in the entire region (6–8). SLN mapping is used to determine whether the tumor has developed lymphatic metastases. If the pathological diagnosis of the SLN is negative, the tumor can be regarded as having no lymphatic metastasis; conversely, it is considered that the tumor has developed lymphatic metastases. The results of the pathological diagnosis of SLNs help determine the prognosis of the disease and guide the choice of the postoperative treatment regimen.

### Advantages of Sentinel Lymph Node Mapping

#### Less Invasiveness and Fewer Postoperative Complications

Sentinel lymph node biopsy technology for patients without lymph node metastases only removes the first group of lymph nodes that drain the tumor. The second group and its posterior lymph nodes are intact, maintaining normal physiological functions, and the short- and long-term complications (9) such as postoperative lymphedema, cellulitis (5), and nerve damage (10) are significantly lower than lymph node dissection (11). In a randomized clinical trial (SENTICOL-2), involving 206 patients with early-stage cervical cancer, 105 people underwent SLN biopsy alone and 101 people underwent only SLN biopsy plus lymph node dissection (12). The results showed that the incidence of postoperative lymphatic system complications was significantly lower in the only SLN biopsy group than that in the lymph node dissection group (31.4 vs. 51.5%; *p* = 0.0046), and the incidence of postoperative neurologic symptoms was also significantly lower than that of lymph node dissection group (7.8 vs. 20.6%, *p* = 0.01) (12).

#### Short Operation Time

Due to the small number of lymph nodes removed and the small anatomical range, the operation time is significantly shorter than that of systemic lymph node dissection, reducing the possibility of wound contamination and the incidence of postoperative infection (13). A retrospective cohort study of 250 cases of endometrial cancer showed that the median time for SLN surgery was 152 min (ranging from 60 to 300 min) and the median time for systemic lymph node dissection surgery was 370 min (ranging from 80 to 600 min) (14).

#### Low Blood Loss

A systematic review of 21 endometrial cancer studies found that sentinel lymphadenectomy was associated with lower estimated blood loss (15). A study of 621 patients with stage I–III endometrial cancer found that the SLN biopsy group had lower blood loss than estimated than the pelvic lymphadenectomy group (median blood loss of 50 vs. 100 ml) (16).

#### Cost-Effective

One study found that an SLN biopsy for medium- and low-risk endometrial cancer ($16,401) was the most cost-effective compared to conventional lymphadenectomy ($18,041) or selective lymphadenectomy based on frozen sections ($17,036). Another systematic review (17, 18) of 21 endometrial cancer studies comparing the costs of three procedures found that no lymph node sampling was less expensive than SLN dissection, which was less expensive than lymph node dissection (15).

In addition, it is very cost-effective to focus resources on advanced pathological examinations (e.g., ultrastaging, immunohistochemistry) for small numbers of SLNs resected by SLN (13).

### Disadvantages of Sentinel Lymph Node Mapping

#### Factors Influencing the Success of Sentinel Lymph Node Mapping and Biopsy

##### Surgeon Proficiency (Learning Curve)

Lymphatic drainage of different organs is complex and SLN localization biopsy is skillful; therefore, the surgeon needs 20–40 practices to master the technique and successfully perform at least 10 SLN operations every year (19–21). During the learning process, the learning curve of the physician’s operational proficiency will affect the success rate of the SLN. The success rate of the SLN can also be influenced by the experience of the surgeon, as a biopsy of suspicious lymph nodes is required.

##### Properties of Different Dyes

In gynecological tumors, a single-center cohort of 218 patients found that indocyanine green (ICG) increased the detection rate of pelvic SLNs compared with blue dye (22). For detection with 99mTc dye, lymph nodes must emit radiation that exceeds a multiple of the preset background radiation intensity to be detected. Some lymph nodes with less than the preset radiation intensity may be missed. In addition, artifacts and background noise can interfere with imaging. The advantages and disadvantages of different tracers will be discussed further later.

#### Risk of Missed Lymph Node Metastases

Since SLN biopsy only determines the status of the first group of draining lymph nodes, a few macrometastases (metastatic tumors >2 mm in diameter), retrograde metastases, jump metastases, and metastase by high endothelial venules (23) may be missed, thereby delaying the optimal treatment of patients and leading to doctor-patient disputes. The reasons for missed diagnoses are common as follows:

##### Stained Part Obscured by Tissue

The visibility of blue dye is worse than that of ICG, and some lymphatic vessels are deep. If covered by thicker tissues, it will affect the recognition of surgeons. However, blue dye is not radioactive or penetrating, therefore such SLNs are often missed in biopsy (24).

##### Tumor Completely Obstructs Lymphatic Vessels

After the metastatic tumor cells proliferate in the SLNs and completely block the lymphatic vessels, the lymphatic vessels lose their function of draining lymph fluid. The tracer fails to flow into this lymph node, which is blocked by tumor cells, leading to misdiagnosis. Clinically, this situation is not uncommon, the lymph nodes occupied by tumors are often extremely enlarged, and pathological sections show “macrometastases.”

##### Diverted Lymphatic Drainage After Organ Removal

When the organ is removed, the lymphatic drainage pathways are gradually occluded and degraded due to the loss of continuous perfusion of lymph fluid from the original organ. A clinical study of early-stage epithelial ovarian cancer found a higher positive rate (88.9 vs. 41.7%) for immediate surgical staging than for delayed surgical staging (25). Results of another clinical trial of early-stage ovarian cancer showed that if SLN mapping was postponed until 5–8 weeks after tumor resection, SLN mapping would not be successfully localized (26).

#### Adverse Reactions

Sentinel lymph node mapping uses exogenous lymph node tracers, carrying the risk of allergic reactions (27, 28) as well as the possibility of ionizing radiation, such as 99mTc, 18F-FDG, and other radioactive tracers. There are many case reports about isosulfan blue, patent blue, and methylene blue (25), often manifested as urticaria, head flushing, bradycardia, skin necrosis, severe respiratory distress, hypotension, and even pulseless electrical activity (29, 30).

### Tracers of Sentinel Lymph Node Mapping Technology

At present, the dyes commonly used in gynecological tumors are blue dyes, indocyanine green, and 99mTc. The tracer injected into the surrounding tumor moves to the SLNs along the same path as the tumor cells. The characteristics of different tracers are shown in Table 1 (31).

**TABLE 1:** Comparison of the characteristics of different tracers.

Characteristic	Blue dyes	ICG	99mTc	References
Affinity	Nucleic acid	Albumin	CD206 (tilmanocept)	(31)
Metabolic process	The kidneys pass urine	The liver excretes	The spleen removes sulfur	(30, 31, 53)
Detection equipment	/	Near-infrared camera	Gamma radiation detector	(42)
Incidence of allergic reaction	High	Low	Low	(4, 18)
	−1.10%	(1/42,000, 0.05%)	(1–6/100,000)	
Price	Low	Middle	High	(12)
Radioactivity	–	–	+	(42)
Reducibility	+	–	–	(31)
Penetrating	–	+	+++	(18)
Peripheral oxygen saturation	Lower	–	–	(18)
Skin staining and necrosis	+	–	–	(4)

*CD206, Mannose receptors on reticuloendothelial cells; ICG, indocyanine green; 99mTc, technetium 99.*

#### Blue Dye

It is widely used and includes the following three types: methylene blue, isosulfan blue, and patent blue. Methylene blue is cheaper than isosulfan blue, which has a higher rate of allergic reactions (1.1%) (19). Methylene blue and isosulfan have a strong affinity for nucleic acids and can stain cancer cells rich in nucleic acids into dark blue (32). The incidence of blue dye allergy was 2%, higher than that of ICG, which was 0.05% (5).

#### Indocyanine Green

Indocyanine green is widely used with high safety, low toxicity, and strong affinity for albumin, and it can be quickly absorbed and distributed in the vascular and lymphatic systems, eventually absorbed and excreted by the liver in the form of a prototype (32, 33). The colorless ICG solution emits green fluorescence under the excitation of the near-infrared light source at a wavelength of about 800 nm (32). Near-infrared light can penetrate tissue 5–8 mm, so even if the lymphatic vessels and nodes are covered by other tissues, the ICG can clearly visualize them (24).

#### Radiotracers

The most frequently used radiotracer for SLN is Technetium 99 (99mTc) labeled colloids which include sulfur colloid, nanocolloid, or calcium phytate (2), requiring a handheld radio-detector to locate lymph nodes after injection. Recent studies have shown that SPECT-CT testing after injection of radioactive dye is more beneficial to the localization of SLN (2). The newly synthesized (99m) TC-Tilmanocept binds tightly to mannose receptors (CD206) in reticuloendothelial cells in lymph nodes, which prevents metastasis to the second group of lymph nodes. The authors suggest that (99m) TC-Tilmanocept is more sensitive than blue dye (34).

#### 18F-FDG

Fluorodeoxyglucose F 18 or 18F-FDG (35, 36) is radioactive, structured like glucose, and easily accumulates in tissues with high sugar consumption, such as tumors. Mapping is required in conjunction with ECT positioning. One clinical study injected 18F-FDG into the cervixes of 20 patients with cervical or endometrial cancer, followed by a dynamic assessment with PET/CT every 5 min within 30 min and PET/CT scans of the radiation profile of pelvic lymph nodes after 60 min to analyze the 18F-FDG uptake pattern (36). It turned out that this technique had a sensitivity of up to 100% for identifying tumor-positive SLNs but a relatively high false-positive (10%) (36). The limiting factor may be the rapid absorption of 18F-FDG into the blood and excretion through the urinary system, affecting the localization of SLN (36). So far, only two articles have mentioned the application of 18F-FDG in detecting SLN.

### Basic Operation Steps

**FIGURE 2:**
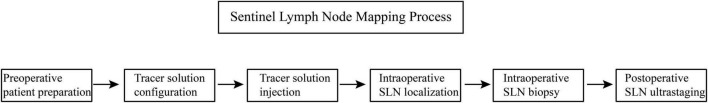
Process of sentinel lymph node mapping. This flow chart shows the process of sentinel lymph node mapping.

The tracer needs to be injected along the lymphatic drainage path to clearly locate the SLNs. Surgery usually requires steps such as patient preparation, tracer configuration, tracer injection, intraoperative SLN localization, intraoperative SLN biopsy, and postoperative ultrastaging (Figure 2).

### Current Application of Sentinel Lymph Node Mapping and Biopsy Technology in Gynecological Malignant Tumors

Recent systematic reviews and meta-analyses (35, 37–41) on SLN mapping technology concerning gynecological malignant tumors are concluded in Supplementary Table 1.

#### Endometrial Cancer

##### Lymphatic Drainage for Endometrial Cancer

The lymph of the uterine corpus is drained into the internal iliac, external iliac, and obturator lymph nodes, and a small amount of the corpus flows into the presacral lymph nodes, eventually converging into para-aortic lymph nodes (6).

##### Sentinel Lymph Node Implementation

###### Injection Site

The localization of sentinel lymph node of endometrial cancer is usually performed at four injection sites: cervix, peritumor endometrial (assisted by hysteroscopy), myometrium/perimetrium, and cervix isthmus (11, 42).

###### Injection Method

Usually, 3–5 ml of 1% solution is injected into the cervix, and within 10–20 min, the dye will accumulate in SLNs (19). Endometrial cancer can be drained directly to the para-aortic area through the adnexal.

###### One-Step Nucleic Acid Amplification

One-step nucleic acid amplification (OSNA) assay is a PCR method in which SLN metastases are detected intraoperatively (43). However, the efficiency of its application in endometrial cancers remains unclear. Referring to pathologic ultrastaging as standard, a systematic review including four studies together with 691 lymph nodes in 237 patients showed that the sensitivity of OSNA is 0.88, specificity is 0.93, and diagnostic odds ratio is 191.23 (43). OSNA seems to be an accurate tool for the intraoperative assessment of SLNs in endometrial cancer.

###### Clinical Studies at the Injection Site

In response to the problem that the injection of tracers in the cervix for endometrial cancer may lead to low detection rates of SLNs in the para-aortic region, a randomized controlled trial of 81 patients with endometrial cancer was carried out. A total of 40 patients were injected with 99mTc at 3 and 9 o’clock in the cervix, and 41 people were injected with it into the endometrium through transcervical catheters (44). The results showed that the detection rate of para-aortic SLN by transcervical endometrial injection was higher (*p* < 0.001) (44). Another randomized controlled study of 151 patients with early-stage endometrial cancer compared the mapping rates of the SLNs by ICG cervical (*n* = 82) or hysteroscopic (*n* = 69) injection (45). The results showed that the mapping rate of endometrial hysteroscopic injection in the para-aortic region was 9.5% higher than that in the cervix, but the difference was not statistically significant (*P* = 0.18) (45). Transcervical injection better identified SLNs in the pelvic region, with a higher overall pelvic detection rate (45) (cervical group 95.1 vs. 76.8% hysteroscopic group) (45).

##### Application of Sentinel Lymph Node Technique in Endometrial Cancer

###### Guide Recommendation

The 2022 edition of the National Comprehensive Cancer Network (NCCN) guidelines has already recommended the application of SLN in endometrial cancer and insists that SLN should precede hysterectomy, and ultrastaging of pathology is strongly recommended as well (46).

The NCCN guidelines indicate that SLN is appropriate for patients with disease confined to the uterus (no metastases on imaging or no significant extrauterine disease detected). For high-grade endometrial cancer, the guidelines suggest that special caution should be paid when performing SLN (46).

###### Clinical Research on High-Grade Endometrial Cancer

Sufficient evidence was found to support the accuracy of SLN biopsy in the staging of high-grade endometrial cancer, and SLN techniques appear to be cost-effective for invasive endometrial cancer (17). One endometrial cancer study showed a sensitivity of 96% and a negative predictive value of 99% for SLN detection of lymph node metastases, showing that SLN and lymphadenectomy were similar in diagnostic accuracy and prognosis for patients with high-grade endometrial cancer (47, 48). A systematic review included data from 429 patients in nine prospective cohort studies to explore the value of SLN biopsy in high-grade endometrial cancer (49). All patients were clinically diagnosed with stage I high-grade endometrial cancers, and all of them were injected with indocyanine green at the cervix to detect SLNs (49). The results showed that SLN biopsy can replace systemic lymphadenectomy and accurately detect lymph node metastasis in patients with high-grade endometrial (49).

###### Clinical Research on the Incidence of Metastasis in Endometrial Cancer

A retrospective study reported on the rates of occult lymph nodes and ovarian metastases in early endometrial cancer successfully mapped by SLN between 2005 and 2018 (50). None of the 510 patients with a non-invasive FIGO grade 1/2 endometrioid carcinoma were found to be SLN positive (50). The incidence of isolated tumor cells increased with an increase in the depth of muscle layer invasion (50). Based on time and cost-effectiveness, it is currently recommended to abandon ultrastaging in patients with non-muscular invasive low-grade endometrial tumors.

#### Cervical Cancer

##### Lymphatic Drainage Pathway for Cervical Cancer

The lymph of the cervix mainly drains into the obturator, internal iliac, external iliac, and common iliac lymph nodes and eventually drains into the presacral lymph nodes and para-aortic lymph nodes (51).

##### Sentinel Lymph Node Implementation

###### Injection Site

The 2022 edition of the Cervical Cancer NCCN Guidelines recommends that (52) the injection site of SLN is 3 and 9 o’clock or 3, 6, 9, and 12 o’clock of the cervix or 45° of rotation at the above four points.

###### Injection Method

About 0.5 ml is injected submucosally each time, with an average activity of 110 MBq in the case of 99mTc (11). Although SLN has been used for tumors >4 cm, but the guideline recommends an optimal tumor size of <2 cm.

###### Ultrastaging

Sentinel lymph node-localized biopsy provides pathological ultrastaging to determine whether radical cervical resection can be performed and whether fertility can be preserved in young patients (11). Although guidelines also recommend SLN ultrastaging, the SLN pathological ultrastaging procedures for cervical cancer need to be further standardized (53).

##### Application of Sentinel Lymph Node Technique in Cervical Cancer

###### Guide Recommended

The 2022 NCCN guidelines recommend that SLN is considered for cervical cancer <2 cm, staging I A1 (with lymph vascular space invasion), IA2, IB1, IB2, and IIA1, which can help reduce the use of lymph node dissection for early cervical cancer (52).

Ultrastaging is strongly recommended to detect a low volume of metastases.

###### Clinical Studies

A study included the data of 928 SLN cases in 313 patients with early-stage cervical cancer from 25 centers to analyze the diagnostic effect of SLN frozen section pathological examination, with conventional pathological ultrastaging set as the gold standard (54). It was found that the sensitivity of SLN in the frozen section through pathological examination was lower (42.3%) and the negative predictive value was higher (89.7%), which could be considered to have a limited diagnostic value (54).

A single-institution study of 75 patients with cervical cancer who underwent SLN biopsy followed by pelvic lymphadenectomy found that lymphovascular invasion significantly reduced the success rate of patient detection (7), with higher inaccuracy in detection (90.9% with lymphatic vascular invasion vs. 41.5% without, *P* < 0.001) and significantly higher lymph node metastasis rate (40.9 vs. 3.8%, *P* < 0.001). It was concluded that, in cervical cancer, the lymphatic vascular invasion has a great impact on the success rate of SLN detection (7).

A study of 103 patients with early-stage cervical cancer (IA1-IB1) who underwent SLN mapping using TC-99m nanocolloid and/or methylene-blue dye and were followed up (8–120 months) found a bilateral detection rate of 83% in all patients, a specificity of 100%, the negative predictive value of 100%, no pelvic or para-aortic lymph node recurrence, and no lower limb edema (55).

A study of 356 patients with stage IA2-IIA2 cervical cancer underwent systematic pelvic lymphadenectomy after SLN biopsy with carbon nanoparticles suspension (56). Results showed that the overall sensitivity was 96.65%, the false-negative rate was 4.35%, and the negative predictive value was 99.29% (56). The sensitivity and negative predictive value were both 100% and the false-negative rate was reduced to 0% for tumors <2 cm (56). It was concluded that the application of carbon nanoparticles suspension in SLN biopsy of early cervical cancer is safe and feasible (56).

An ongoing multicenter clinical trial to compare 3-year disease-free survival and quality of life after cervical cancer SLN or SLN + PLN (pelvic lymph node dissection) has enrolled 950 patients of IA1-IIA1 cervical cancer with lymphovascular invasion starting in the second quarter of 2018 and to be followed up until the second quarter of 2026 (57). The results of this study will help reveal whether SLN technology is superior to PLN in disease-free survival and health-related quality of life in early cervical cancer.

#### Vulvar Cancer

##### Lymphatic Drainage Pathway for Vulvar Cancer

The lymph of the vulva flows primarily to superficial inguinal nodes and then drains into deep inguinal/external iliac nodes (58).

##### Sentinel Lymph Node Implementation

The 2022 edition of the NCCN guidelines recommend injecting a total of 4 ml of dye at 4 sites in the 2, 5, 7, and 10 o’clock directions of the tumor (59).

##### Application of Sentinel Lymph Node Technique in Vulvar Cancer

###### Guide Recommendation

In the 2022 NCCN guidelines for vulvar cancer, SLN is recommended for T1b or T2 vulvar cancer patients with negative inguinal lymph nodes and no history of vulvar surgery (59). Subsequent treatment options are selected according to the size of the metastasis in positive SLN. Unilateral SLN may be used for primary tumors of <4 cm diameter and locating tumors ≥2 cm from the midline; if locating <2 cm, bilateral SLN should be performed. Pathological ultrastaging is equally necessary.

###### Clinical Studies

One study compared SLN detection in vulvar squamous cell carcinoma (VSCC) using ICG + 99mTc nanocolloids and blue dye + 99mTc nanocolloids (24). The results showed that 65.3% (32/49) of the cut lymph nodes were stained blue in the blue composite dye group, and 92.5% (49/53) of the cut lymph nodes in the ICG composite dye group showed green fluorescence (24) suggesting that ICG + 99mTc nanocolloids were found to be superior for visual inspection of SLNs in patients with VSCC (24). In addition, the blue dye can also discolor the vulva, resulting in inaccurate SLN positioning, obstructing the normal surgical field of vision, and causing larger surgical incisions and more postoperative complications (24). Therefore, ICG is recommended to be popularized.

In a study of 173 patients with focal invasive vulvar squamous cell carcinoma <4 cm, the sensitivity of intraoperative frozen SLN sections was 89.7%, the specificity was 99.5%, the positive predictive value was 97.2%, and the negative predictive value was 98.2% (60). It was concluded that intraoperative frozen sections can accurately assess the need for further inguinal lymphadenectomy (60).

A review of 65 articles on the clinical use and technical procedures of SLN biopsy for vulvar cancer found that SLN negative was associated with low inguinal recurrence and good disease-specific survival at 5 years (21). SLN biopsy is more cost-effective than lymphadenectomy in early-stage vulvar cancer and is currently the standard treatment for women with negative lymph nodes (21).

##### Application of Imaging Methods in Sentinel Lymph Node of Vulvar Cancer

In vulvar cancer, 18F-FDG-PET/CT SLN accuracy is less than 75%, and MRI sensitivity is about 50% (11).

The sensitivity and reliability of ultrasound in vulvar cancer need to be further studied.

In one study, SLNs were identified by contrast-enhanced ultrasound (CEUS) (61). Ultrasound examination of SLN in the groin-femoral area was performed immediately after injection of contrast agent, with a sensitivity of 81.2% (61). In addition, other studies have shown that ultrasonography can detect octagonal metastases that might be missed by conventional pathologic examinations (17).

##### Effect of Sentinel Lymph Node on Prognosis

In the GROINSS-V-II clinical trial of early vulvar cancer with a diameter <4 cm, only 1.6% of SLN metastases with a diameter <2 mm were relapsed 2 years after 50 Gy radiotherapy, but 22% of metastases with a diameter >2 mm were relapsed 2 years after 50 Gy radiotherapy (62). It can be concluded that 50 Gy radiotherapy can replace lymph node dissection for SLN <2 mm metastases, but not for metastases >2 mm.

A study of 111 patients with early-stage vulvar cancer found that 22 had positive sentinel nodes and 89 had negative sentinel nodes (63). Recurrence was found in 44% (7/22) of lymph node-positive patients and in 8% (6/74) of lymph node-negative patients who were followed up for at least 1 year (63). Among 6 patients who had a recurrence, 2 patient’s pathology examinations were not carried out in strict accordance with the requirements of the protocol, leading to misdiagnosis and small metastatic lesions being undetected, indicating the importance of pathology in SLN biopsy of vulvar cancer (63).

In a nationwide analysis of 286 patients with stage IB-II single-focal <4 cm of vulvar squamous cell carcinoma who underwent SN surgery in Denmark, 23 of 190 patients who were negative for SLNs (66.4%) developed varying degrees of recurrence during 1–83 months of follow-up (64). Disease-specific survival was 93% for the 190 patients and overall 3-year survival was 84% (58% for relapsed patients) (64). It was concluded that the patients with early vulvar cancer are safe after SLN operation, and the recurrence rate is relatively lower (64).

#### Ovarian Cancer

##### Lymphatic Drainage Pathways for Ovarian Cancer

There are 3 drainage routes for ovarian cancer (65), which are along:

1. Infundibulopelvic ligament (suspensory ligament) – para-aortic/paracaval lymph nodes;
2. Ovarian ligament – internal iliac/obturator lymph nodes;
3. Round ligament – inguinal lymph node (66).

##### Sentinel Lymph Node Implementation: Injection Site

Commonly used SLN dye injection sites select ovarian ligament stumps, and infundibulopelvic ligaments and the ovarian cortex is not selected because of the low detection rate and the risk of tumor rupture (67). Two surgical videos demonstrate the process (68) of pelvic and para-aortic SLN biopsy for laparoscopic ovarian cancer, recommending that the malignancy be removed along with draining lymphatic vessels, removing at least the first 2 SLNs in each pathway (69).

##### Application of Sentinel Lymph Node Technique in Ovarian Cancer

The NCCN guideline (2022 edition) has not yet been recommended.

A systematic review selected 145 patients in 10 studies and found SLN detection rate to be 90.3% (70). Another systematic review included 10 studies with a total of 179 patients, with a total SLN technology detection rate of 87.7% (10). In the cases of lymph node metastases, the sensitivity of SLN surgery was 90.9% and the negative predictive value was 98.8%. SLN surgery appears to be feasible and safe, reliably determining the status of lymph nodes in patients with early-stage ovarian (10).

In a clinical trial combining 99mTc and ICG to detect SLN in clinical stages 1 and 2 ovarian cancers (67), 20 patients, including 5 who had previously undergone hysterectomy and were unable to receive uterine-ovarian ligament (67), were selected. The test injection sites were the pelvic funnel ligament and the uterine-ovarian ligament stump, and the results showed that 93% (14/15) of the pelvis and 100% (20/20) of para-aortic SLN were detected, with no SLN-related complications occurring during 30 days of follow-up after surgery (67). Para-aortic and pelvic metastases were found in 19 patients, of whom only 1 had para-aortic lymph node metastases (67).

One clinical trial used intraoperative intravenous ICG to detect lymph nodes (LNs) metastatic in advanced ovarian cancer (71). The ratio of tumor to background fluorescence (TBRs) was automatically calculated, and the cutoff TBR was 1.3 (71). The univariate analysis showed that the fluorescence ratio was associated with pathological malignancy (*P* = 0.03), and the fluorescence intensity is higher in malignant lymph nodes invaded by tumor cells and lower in benign lymph nodes (71). It was concluded that *in vitro* ICG fluorescence imaging after intravenous ICG injection has a good sensitivity (80%) for the detection of retroperitoneal positive LNs of advanced ovarian cancer, but its specificity (41%) is not enough for differentiating benign and malignant lymph nodes, and its negative predictive value is high, which can assist the pathological analysis of lymph node specimens (71).

In one study to determine the role of SLN ultrastaging in early-stage ovarian cancer, 30 patients underwent SLN mapping and pathological ultrastaging with a slice thickness of 200 microns (72). TC-99m and ICG detection rates were high, reaching 30/30 (100%) and 28/30 (93.3%) (72). Six people were upgraded after ultrastaging, of which two people found that macrometastases previously failed to be undiscovered, and four patients found implantation in other areas (omentum, fallopian tube, Douglas cavity, etc.) (72). It was concluded that a uniform ultrastaging protocol was critical for low-volume metastasis detection and for providing repeatable information for future studies (72).

The number of completed and published clinical studies of ovarian cancer is small, and their accuracy and clinical applications require larger multicenter studies.

## Future Perspectives

### The Advantages of Sentinel Lymph Node to Help Clinical Work in the Future

#### Assist in the Diagnosis of Pathology

Clinical studies of advanced ovarian cancer have shown that the negative predictive value of ICG intravenous injection is 99% and can be used to assist in pathological diagnosis (71).

#### Repair of Lymphedema of Lower Extremities

Sentinel lymph node techniques can be used to identify target lymph nodes intraoperatively and then perform lymphatic venous anastomosis to repair lower extremity lymphedema (61).

#### Predict Prognosis

It was found that, when SLN was injected with a mixture of blue dye and carbon particles, the blue dye passed quickly through the SLN, while the carbon particles remained near the SLN tumor cells (73). Therefore, the presence of carbon particles can determine whether lymph nodes have metastasis. In addition, in advanced ovarian cancer, lymph nodes were dissected and observed under a fluorescence microscope after intravenous injection of ICG, and higher fluorescence intensity was found in lymph nodes invaded by malignant tumor cells (71). In the mouse model of melanoma, the uptake of 18F-FDG in lymph nodes with positive tumor metastasis was significantly different from that in normal lymph nodes, allowing the identification of macrometastases (36). Hence, we may distinguish normal lymph nodes from malignant lymph nodes by fluorescence intensity and uptake pattern of 18F-FDG.

#### Guidance of Adjuvant Therapy

Recent studies have shown that a large number of substances, such as VEGF-A, VEGF-C, VEGF-D, and TGF-β, have been produced by tumors long before the occurrence of lymphatic metastasis, which changes the microenvironment of lymph nodes and affects the state of immune cells (74). Injection of immune activator or immunosuppressant decoy receptor in SLNs may activate immune cells, activate anti-tumor immunity, and resist immunosuppression to prevent tumor metastasis and spread. In addition, the injection of radio chimeric antibodies and targeted chemotherapy drugs may maximize the tracking and killing of tumor cells along the tumor metastasis pathway, while minimizing the damage to normal cells.

In short, with the application of more new technologies and new methods, SLN technology will be able to help reduce surgical accidental injuries, repair lymphedema of the lower limbs, assist in pathological diagnosis, treatment, and prognosis, and facilitate the precise treatment of gynecological tumors.

### Disadvantages of Sentinel Lymph Node Mapping to Be Improved

#### Reduce False Negatives

##### Unilaterally Mapping Is Replaced by a Bilateral Mapping

Studies have shown that, when SLN is detected bilaterally, the detection rate of LN metastases increases and the false-negative rate decreases compared with unilateral SLN (13).

##### Complete Resection of UPLT

Upper paracervical lymphovascular tissue (UPLT) contains the SLN of cervical cancer and can be resected and dissected in a short period with no risk of autonomic nerve injury; it is located between the tail of the superior vesical artery and the ventral ureter and between the broad ligament and the occluded umbilical artery (75). One study performed pelvic lymphadenectomy in 145 patients with stage IA1-IB1 cervical cancer and no other intraoperative complications were observed (75). The results showed that 2% of women had isolated lymph node metastases in UPLT, leaving a risk of recurrence (75). Isolated lymph node metastases were detected in UPLT in more than half of the subjects, indicating the importance of studying this tissue (75). Surgical excision of UPLT requires little additional operative time, carries a negligible risk of surgical complications, and does not damage the autonomic nerve (75). Therefore, total excision of the UPLT tissue should be performed as part of SLN for cervical cancer.

#### Application of New Mapping Methods

At present, commonly used SLN localization methods mainly use the naked eye (blue dye), radionuclide detection (99mTc), and near-infrared (ICG). Since the magnetic field has no ionizing radiation on the human body, development of superparamagnetic iron oxide nanoparticles and their derivatives and the application of magnetic dyes in gynecological tumors should be accelerated (32, 76–78).

#### Development of New Tracers

The ideal dye should have high light stability, solubility, and fluorescence quantum yield (79), which can accurately indicate the location of the SLNs during the operation and be completely cleared by the body after the operation. The dye should be non-toxic and have minimal side reactions.

#### Biodegradable Tracer

Nanocarbon particles remain in monocytes in the reticuloendothelial system of lymph nodes, and the blue dye stains the skin (32); therefore, it is necessary to develop more biodegradable dyes. Biodegradable micromagnetic particles may be the future direction of research and development.

#### Tumor-Targeted Tracers

Currently, SLN technology can only localize lymph nodes but cannot judge their status, and the judgment of lymph node status depends on pathology. In the future, it will be necessary to develop non-invasive methods to determine tumor metastasis without removing lymph nodes during surgery. A study identifying sentinel and metastatic lymph nodes by intravenous infusion of the tumor-targeted tracer Panitumumab-IRDye800CW found that the median mean fluorescence intensity of lymph nodes with tumor metastasis was significantly higher than that of benign lymph nodes (80). Moreover, the analysis of the 5 lymph nodes with the highest fluorescence intensity in each patient showed 100% sensitivity, 100% negative predictive value, and 85.8% specificity, indicating that tumor-targeted tracers may be the development direction of SLN tracers in the future (80).

In conclusion, SLN technology has great development space at present. More scientific exploration and clinical trials are needed to improve the sensitivity and specificity of SLN technology, reduce the false positive rate and false-negative rate, simplify surgical procedures, and reduce adverse reactions.

## Author Contributions

CW and TW conceived the topic and wrote the first draft. YX and WS reviewed the manuscript, tables, and images. All authors revised and approved the final draft.

## Conflict of Interest

The authors declare that the research was conducted in the absence of any commercial or financial relationships that could be construed as a potential conflict of interest.

## Publisher’s Note

All claims expressed in this article are solely those of the authors and do not necessarily represent those of their affiliated organizations, or those of the publisher, the editors and the reviewers. Any product that may be evaluated in this article, or claim that may be made by its manufacturer, is not guaranteed or endorsed by the publisher.
