# Supplementary material for: Sentinel Lymph Node Mapping: Current Applications and Future Perspectives in Gynecology Malignant Tumors
Source: Front Med (Lausanne). 2022 Jun 29;9:922585. doi: 10.3389/fmed.2022.922585 (PMC9276931; doi:10.3389/fmed.2022.922585)
Supplement: Supplementary file 1 [file Table_1.pdf]

Supplementary Table 1: **Systematic reviews and meta-analyses on sentinel lymph node mapping technology in gynecological tumors**

**Main research topic**	**Tumor types**	**Publishing date**	**Number of studies included**	**Number of pooled studies**	**Main results**	**Reference**
Diagnostic performance of 18F-FDG PET/CT for preoperative lymph node staging	Vulvar Cancer	2021 Feb	10	169 (A qualitative analysis)	Sensitivity: 76%, specificity: 88%, NPV: 92%, PPV: 70%. PET/CT is recommended for T2 or larger tumors, with metastases suspected. Groin metastases may be excluded by negative whole-body PET/CT results.	[34]
Diagnostic value of indocyanine green fluorescence guided sentinel lymph node biopsy	Vulvar Cancer	2021 May	13	380	SLN detection rate: 89.7 to 100%.	[36]
Validity of ICG dye in detecting SLNs preoperatively	Cervical Cancer	2018 Dec	8	661	Bilateral and unilateral detection rates: ICG higher	[37]
Compare the detection rate of fluorescent agents to blue dye and/or radio-colloid for the SLN	Breast, Gynecological and Dermatological Cancer	2020 Nov	All: 55, (Gynecological: 13)	>7200	ICG provides higher detection rate and detects more SLNs than blue dye and radio-colloid in each patient; the pooled risk difference between blue dye and ICG: 0.17; mixed models show a significant correlation between ICG dosage and both bilateral and overall SLN detection.	[38]
Assess the differences in bilateral and overall SLN detection rates with ICG vs. combined (99mtc & blue dye)	Cervical Cancer	2022 Jan	7	589	Pooled overall SLN detection rate found no significant differences	[39]
Utility of intraoperative fluorescence imaging	Gynecologic Cancer	2021 Jun	74	Not found	ICG is safe, reliable, feasible, and time-efficient, offering higher bilateral detection rates than other tracers. Limitation: body mass index. Cystoscopic intraureteral application decresaes the risk of injury.	[40]
